# Comment on and reply to "Analysis of variation of amplitudes in cell cycle gene expression" by Liu, Gaido and Wolfinger: On the analysis of gene expression during the normal, eukaryotic, cell cycle

**DOI:** 10.1186/1742-4682-2-47

**Published:** 2005-11-18

**Authors:** Stephen Cooper

**Affiliations:** 1 Department of Microbiology and Immunology, University of Michigan Medical School, Ann Arbor, Michigan 48109-0620, USA

## Abstract

**Background:**

The paper of Liu, Gaido and Wolfinger on gene expression during the division cycle of HeLa cells using the data of Whitfield et al. are discussed in order to see whether their analysis is related to gene expression during the division cycle.

**Results:**

The results of Liu, Gaido and Wolfinger demonstrate that different inhibition methods proposed to "synchronize" cells lead to different levels of gene expression. This result, in and of itself, should be taken as evidence that the original work of Whitfield et al. is flawed and should not be used to support the notion that the cells studied were synchronized or that the microarray analyses identify cell-cycle-regulated genes. Furthermore, the DNA content evidence presented by Whitfield et al. supports the proposal that the cells described as 'synchronized' are not synchronized. A comparison of the gene expression amplitudes from two different experiments indicates that the results are not reproducible.

**Conclusion:**

It is concluded that the analysis of Liu, Gaido, and Wolfinger is problematic because their work assumes that the cells they analyze are or were synchronized. The very fact that different inhibition methods lead to different degrees of gene expression should be taken as additional evidence that the experiments should be viewed skeptically rather than accepted as an approach to understanding gene expression during the cell cycle.

## Introduction

The recent paper by Liu, Gaido, and Wolfinger entitled "Analysis of Variation of Amplitudes for Cell Cycle Gene Research" [1] requires comment. Because the subject of gene expression variation during the cell cycle is such an important topic of current interest, it is necessary that any work supporting cycle-specific gene expression be beyond reproach and criticism. If the paper by Liu, Gaido and Wolfinger [1] remains unchallenged, it will merely be used as another reference supporting the data of Whitfield et al. [2] regarding gene expression variation during the division cycle. Because I believe that such a conclusion is unwarranted, I now summarize my objections to this analysis so that readers may be able to compare two alternative views of the cell cycle and gene expression during the cell cycle.

As the reader will gather, the view I present here is not widely held, and is even a minority viewpoint. But I hope that readers will at least agree that science does not proceed by majority vote. The most important point I make is a critique of the fundamental paper analyzed by Liu, Gaido and Wolfinger [1], that of Whitfield et al. [2]. I point out that the two experimental approaches analyzed, experiments 2 and 4 of Whitfield et al., are not analyses of synchronized cells. I propose that the cells studied in the Whitfield et al. paper are not synchronized – at all. They may be "aligned" for certain cell properties, and even this may be problematic. But as will be pointed out in detail below, such alignment is not equivalent to synchronization.

## Theoretical critique of whole-culture synchronization

The two Whitfield et al. experiments [2] analyzed by Liu, Gaido and Wolfinger [1] are a double thymidine block experiment (thy-thy), and a thymidine-nocodazole block (thy-noc). I propose is that a truly synchronized culture is one where the starting cells are all of the same genome content and cell size and thus reflect the properties of cells of a particular cell-cycle age while growing in unlimited medium. If one does not narrow the size distribution, or does not have the starting cells mimic a particular cell (with respect to DNA content, cell size and cell composition) of a particular age, then the cells are not synchronized. A complete analysis of this idea has been presented [3-16] and I merely refer the readers to these papers. (Some of these papers can be read at .)

## Criteria for cell synchronization and analysis of gene expression

One should apply clearly stated and stringent criteria for synchronization and microarray experiments. Such criteria are listed here so the reader can at least see why it is proposed that the Whitfield et al. experiments are problematic.

### Criteria for successful synchronization

1) If newborn cells are produced by the synchronization method, there should be a minimal increase in cell number for a period of time covering a significant fraction of the interdivision time.

2) The rise in cell numbers during division should occur over a relatively small fraction of the total interdivision time. It may be as small as 10% for 90% of the final rise in cell number, or it may be as large as 20–25%. Knowing this value is important in judging a synchronization procedure.

3) At the time of synchronous division, the cell number should double. If cell number does not double, that means some cells are dead or altered; this minority of cells could be giving results that obfuscate the results emanating from the majority of dividing cells.

4) There should be at least two successive cycles available for analysis. If only one cycle is analyzed, the results may merely reflect artifacts or perturbations resulting from synchronization. Presumably, but not necessarily, these artifacts would be eliminated in the second cycle.

5) Successive generations (i.e. the time between rises in cell number) should be of equal length and equal to the doubling time of cells in exponential growth.

6) Data points should show synchrony without any need to connect points or draw a suggestive line indicating synchrony. The data should speak for themselves.

7) The DNA distribution of cells should be narrow in the synchronized cells and these distributions should then reflect the movement of cells through the division cycle. Thus, newborn cells should be essentially pure cells with a G1-phase amount of DNA, the DNA content should then move through S-phase contents, there should be a period of time when cells have only G2-phase DNA contents, and then there should be a return to essentially pure G1-phase DNA contents.

8) The size distribution of newly synchronized cells should be narrower than the size distribution of the original population, cell size should increase as the cells move through the cell cycle, and during the period of cell division there should be a bimodal distribution of cell sizes.

9) Cell numbers should be determined by a method that eliminates investigator bias. For example, electronic cell counting is to be preferred to microscope counting chambers.

10) Only selection methods can give synchrony. Whole-culture methods, using inhibition or starvation, cannot synchronize cells. This is not so much a criterion as a theoretical rule regarding synchronization in general.

11) Alignment of cells so that cells all have a particular property in common (e.g. all cells have a G1-phase DNA content) does not mean that the cells are synchronized. Synchronized divisions are the *sine qua non *of synchrony.

### Criteria for successful analysis of gene expression during the division cycle

12) Gene expression results should be replicated (with allowance for normal synchrony decay) in successive cycles. If data do not repeat over two or more cycles, the cells are very likely perturbed by the synchronization method.

13) Peaks in gene expression should decay when expression is studied over more than one cycle. This is because synchrony, if normal and unperturbed, should decay.

14) If a selection method is used, a mock selection should be performed where the selection procedure is performed but the cells are all recombined together and analyzed. These combined cells should not give a variable pattern of gene expression. This controls for perturbation of the culture by the selection method.

15) Results using different synchronization methods should give the same results. Different experiments should be reproducible in cyclicity and in phasing, and thus independent of synchronization methods. That is, the results should not depend on the particular synchronization method used.

### Criteria for successful use of microarrays to analyze cycle-related gene expression

16) Analyses should be performed more than once, and the results should be "reproducible". The qualification on reproducible is related to the acceptance of some degree of statistical variation.

17) The data should be published in accordance with the MIAME (Minimum Information About a Microarray Experiment) or MAGE (microarray gene expression object model) standards, so that the public data can be analyzed.

18) Microarray results should be compared to randomized data to show that the observed cyclicities are not the result of random noise or experimental variation. Satisfaction of this criterion, however, does not mean the results are necessarily related to the cell cycle, as perturbations of cells by a synchronization procedure may still be present.

19) Criteria for successful identification of cyclicity should be determined before microarray analysis.

20) Both false positives and false negatives should be considered in the analysis. Just because a particular gene result fits pre-existing data collected by classical means, one must not consider this to support the results unless the previous synchronization method is different. Otherwise the microarray experiment just repeats the same experiment, with a repetition of the artifacts of synchronization in two independent experiments.

21) If some genes are expressed differently in two successive cycles this should invalidate the entire experiment – even for those genes that are expressed similarly in two successive cycles – because the non-repeating patterns indicate that there are artifacts produced by the synchronization.

## Are the HeLa cells analyzed truly synchronized?

If one looks at the experiments of Whitfield et al. [2] it is clear that the cells are not synchronized. The DNA patterns presented by Whitfield et al. testify to the fact that the cells are not only not synchronized but are also perturbed. The DNA patterns in the thy-thy and thy-noc experiments are quite inconsistent with the proposal that the cells are synchronized. The flow cytometric data on DNA contents during growth of the arrested cells shows the cells are not synchronized. The DNA patterns start with an 8N (it appears) value for DNA content, which goes down to 2N and back to 4N and never repeats the 8N. In between the start and the end there is no systematic variation of cells through G1, S and G2.

In summary, the work of Whitfield et al. is not a synchrony experiment, and the results in the paper [2] show the cells are not synchronized. What we have here is a perturbation experiment. If one wishes to look at the response of genes to perturbation, then it is a good experiment. The problem is whether the methods of Whitfield et al. lead to an understanding of what happens in the normal, unperturbed cell. Theory says they are not synchronized and experimental results from the Whitfield et al. paper itself support this proposal.

## Variation in gene expression: meaning and relevance to the cell cycle

It is of interest to look at the basic data in Table 1 of Liu, Gaido and Wolfinger [1]. In Table 1 they present results on the gene expression amplitudes in two different experiments. In Table 1 they are trying to explain differences between different experiments in terms of variation in the phase angles of peaking of gene expression in different individual cells. Thus, given a particular variation in gene expression in different cells, one can account for different amplitudes based on the variation in phase angle of the peak of expression. If one had a particular cell growing with a 20 hour doubling time, and in some cells the peak was at 9 hours, others 10 hours, and other 11 hours, the amplitude would be reduced based on the distribution and frequency of the peak of expression in different cells.

At this point I have philosophical viewpoint to express, which should, at a minimum, be made explicit. I propose that for a given cell, if there is a particular pattern of gene expression during the cell cycle (something, I may add, that I am skeptical of at the start – that is, I am skeptical of whether there are a large number of genes with cell-cycle dependent expression), then it is the object of an experiment to know what that pattern is. Thus, given a single cell growing in an unlimited supply of fresh medium, if there was a sinusoidal expression in this cell that had a certain amplitude and a certain time of peaking, a good experiment would get this result. Two different true synchronization experiments should lead to the same results.

In the Liu, Gaido and Wolfinger paper the authors talk about comparing "different conditions". I apologize for being so fastidious and demanding, but I want to distinguish between what I consider different "conditions" and different "experimental approaches" to determining something about cells. For example, "different conditions" means growing cells in one case at 25°C and in another case at 37°C and seeing what happens to them or what is different in these different conditions. Or one may have Medium A and Medium B and compare the cells for some property. These are "different conditions." But when you take the same cells, growing in a given single condition, and then try to analyze them using two different methods, what you should get, in an ideal world, are results that agree with each other. To summarize, the Whitfield et al. experiments are using different methods of analysis, not "different conditions."

I don't know how people measure the speed of light, but I do remember that there are many different approaches. But when all is said and done, all of these methods give rather reproducible results. That is, different analytical "methods" give the same result. I propose that we should analogize the thy-thy and the thy-noc to different methods examining same condition.

**Figure 1:**
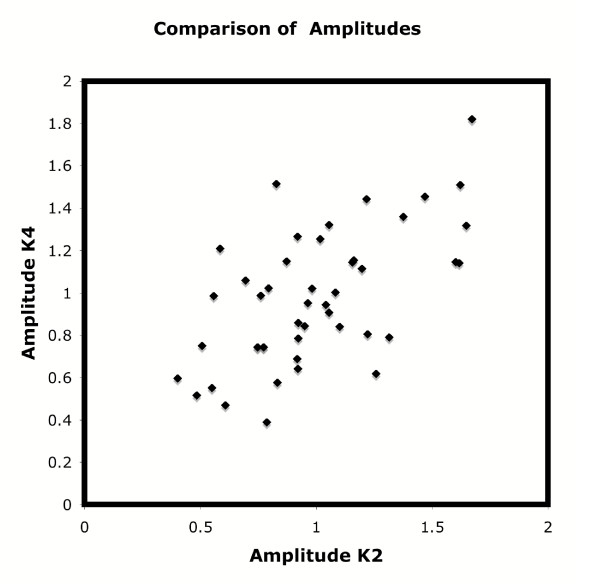
Replotting of the K values of the two experiments analyzed by Liu, Gaido and Wolfinger [1].

Returning now to the comparison of the two experiments, I present a graph (Figure 1) of the K values in a scatter diagram. The R2 value is 0.37, which some may say is "rather strong" and others may say is "rather weak". In measuring the size of an atom, this value would be rather weak. In sociological measurements the data would be considered rather strong. There is no absolute measure of how one should accept the scatter as being strong. For a person who is inclined to believe, the data, if correct, should show a clear 45 degree line from lower left to upper right. For a person who says that the results of the two Whitfield et al. experiments are not reproducible, the data allow that. I look at the data in this scatter diagram and see non-reproducible results.

Figure 1, in a sense, is a redoing of the Figure 3 of Liu, Gaido, and Wolfinger [1] in a more intuitive manner. What Liu et al. are saying is that if one has broad enough error bars on the data one can say that many of the points do have similarity or even "identity". Perhaps. But that may be, and in my view is, a judgment call.

One important point that I wish to bring up is that if the two experiments have "different strengths of synchronization", then one would expect that one would have a systematic difference in amplitudes. Thus, if synchrony were sharper in one than the other, the amplitudes would be higher in that experiment than in the broader synchronization. This does not appear to be the case, which is again support for the notion that one should be wary of accepting the results of Whitfield et al. as a synchrony experiment.

## Summary

The work of Liu, Gaido and Wolfinger [1] is a statistical analysis of a well-regarded paper [2] on the pattern of gene expression during the cell cycle of eukaryotic cells. There are no fundamental problems with the statistical analysis, but there are problems with the underlying experiments that form the data base for analysis.
